# Computational aptamer design for spike glycoprotein (S) (SARS CoV-2) detection with an electrochemical aptasensor

**DOI:** 10.1007/s00253-024-13066-w

**Published:** 2024-03-12

**Authors:** Alessia Cossettini, Laura Pasquardini, Antonello Romani, Aldo Feriani, Debora Pinamonti, Marisa Manzano

**Affiliations:** 1 Department of Agriculture, Food, Environmental and Animal Sciences, University of Udine, Via Sondrio 2/A, 33100 Udine, Italy; 2 Indivenire Srl, Via Sommarive 18, 38123 Trento, Italy; 3 Arta Peptidion srls, Via Quasimodo 11, 43126 Parma, Italy

**Keywords:** Spike protein, Aptamer, Screen-printed electrodes, Biosensor, Bioinformatic platform

## Abstract

**Abstract:**

A new bioinformatic platform (APTERION) was used to design in a short time and with high specificity an aptamer for the detection of the spike protein, a structural protein of SARS-CoV-2 virus, responsible for the COVID-19 pandemic. The aptamer concentration on the carbon electrode surface was optimized using static contact angle and fluorescence method, while specificity was tested using differential pulse voltammetry (DPV) associated to carbon screen-printed electrodes. The data obtained demonstrated the good features of the aptamer which could be used to create a rapid method for the detection of SARS-CoV-2 virus. In fact, it is specific for spike also when tested against bovine serum albumin and lysozyme, competitor proteins if saliva is used as sample to test for the virus presence. Spectrofluorometric characterization allowed to measure the amount of aptamer present on the carbon electrode surface, while DPV measurements proved the affinity of the aptamer towards the spike protein and gave quantitative results. The acquired data allowed to conclude that the APTERION bioinformatic platform is a good method for aptamer design for rapidity and specificity.

**Key points:**

• *Spike protein detection using an electrochemical biosensor*

• *Aptamer characterization by contact angle and fluorescent measurements on electrode surface*

• *Computational design of specific aptamers to speed up the aptameric sequence time*

**Supplementary Information:**

The online version contains supplementary material available at 10.1007/s00253-024-13066-w.

## Introduction

The growing demand for specific molecule detection has stimulated the development of new strategies for rapid and sensitive identification of various targets. Research has focused on single-strand DNA (ssDNA) sequences as selective bio-recognition elements for biological applications in various fields from clinical, environmental and food analyses.

DNA sequences (25–90 nt) called aptamers (from Latin *aptus* and Greek *meros*) can assume secondary and tertiary structure and can be used for specific detection of molecules of different nature, from ions to whole cell (Manzano 2021). Last years, attention has been pointed on clinical application of aptamers for the creation of aptasensors able to detect specific biomarkers in body fluids like plasma, serum and saliva.

The pandemic, due to the spread of SARS-CoV-2 developed in 2020, requested the development of new strategies for fast and specific detection of the virus not only in biological fluids, but also in other kind of samples, from surfaces, food, water, etc. The capability of aptamers to specifically bind proteins has promoted the design of aptamers to detect the presence of SARS-CoV-2 using as target the spike (S) protein.

The aptamer immobilized on the sensing surface of the sensor can be used as a detection probe to build an aptasensor (Li et al. 2019; Pasquardini et al. 2022) which can ensure specific detection. The reduction of the sample and reagents volume leads to consequent reduction of costs. Among the different transducers, electrochemical biosensors have shown high versatility, easiness to use and low cost compared to other devices making them useful for screening purposes.

The aptamer technology is emerging as attractive alternative to monoclonal antibodies. Indeed, its efficiency and widely applicability, in addition to an impressive target binding selectivity and affinity and low immunogenicity, make aptamers ideal recognition elements for use also as therapeutics. Other advantages include high stability, long shelf-life and rapid tissue penetration based on the relatively small molecular weights (Eyetech Study Group 2002) (Oney et al. 2009). Finally, the rapid improvement of automated nucleic acid synthesis occurred in the last few years enables easy, cost-effective chemical synthesis and modification of functional moieties, as well as large-scale commercial production (Zhu et al. 2012). Moreover, owing to their three-dimensional conformation, aptamers can reach very high specificity and binding affinities (K_D_s usually ranging from picomolars to nanomolars), this being comparable or even stronger than monoclonal antibodies (Song et al. 2012a, b).

The classic selection of aptamers is obtained by the SELEX method (systematic evolution of ligands by exponential enrichment) (Ellington and Szotok 1990), a long and time-consuming method which takes at least 3–4 months (selection & sequencing) and more than $5500 to obtain specific aptamer candidates with low hit rates. To effectively reduce costs and times an integrated technological platform (APTERION), property of Arta Peptidion, (info@artapeptidion.it), based on artificial intelligence (AI) whose core unit (Classification Unit) is represented by hybrid neural networks, was developed to implement the Computational Selex. With this approach, an aptamer can be designed, selected and hence synthetized in up to 3 weeks thanks to the computational protocol which resembles the experimental one.

The aim of this work was to evaluate the applicability of an aptamer selected through the APTERION platform to specifically bind the SARS-CoV-2 spike protein. Contact angle measurements and fluorescence spectroscopy and microscopy have been used to characterize the aptamer binding on the surface of the electrodes. Electrochemical measurements have also been used to verify the interaction between the selected aptamer and the spike protein and to test the aptamer specificity against competitors.

## Materials and methods

### Materials and instruments

Phosphate buffer saline (PBS) 1X (pH 7.4), bovine serum albumin (BSA), and TRIS Buffer (50 mM Tris–HCl, 2 mM MgCl_2_, 150 mM NaCl pH 7.5) were purchased from Sigma-Aldrich (Milan, Italy).

The aptamer AP_7462DNA, 5′-GCGGCGCGGTATGGAATTAGTGACCTTCCGCGCGCCCCATTTTTTATAGGGGCCGC-3′, was synthetized by Metabiom international AG (Planegg, Germany) and used for the electrochemical tests.

The aptamer with the addition at 5′ end of C6 S–S-polyT and a 6-Carboxytetramethylrhodamine (TAMRA) at 3′ end (named aptamer-TAMRA) was purchased from GenScript Biotech (Leiden GenScript Biotech, Leiden) and used for the contact angle and fluorescence experiments.

A solution of potassium ferrocyanide (K_4_[Fe(CN)_6_ × 3H_2_O]) 10 mM prepared using sterilized PBS buffer 1X (AnalytiCals, Milan, Italy) was used as redox probe in the electrochemical tests.

The electrochemical analysis was conducted using an Potentiostat/Galvanostat/Impedance Analyzer (Metrohm Italia, VA, Italy) and screen-printed carbon electrodes (110, Metrohm Italia, VA, Italy).

### Aptamer design

The design of the aptamer was obtained by an appropriate bioinformatic platform (APTERION), property of Arta Peptidion (Parma, Italy), which generates (design unit) and select (classification unit) the best aptamer sequences for target proteins (any screening analyses of aptamer sequences will be provided, upon (motivated) request, by Arta Peptidion (info@artapeptidion.it)).

The training dataset of complexes aptamer-protein was built over experimentally validated complexes, including Aptamer Base and Protein Data Bank (PDB). These are separated into training and test datasets. To feed aptamer and protein sequences into our classification model, both sequences were encoded into a numerical representation with an optimal encoding function for protein and aptamer sequences. The encoding method was implemented in Python scripts.

The aptamer sequences were designed by using Arta Peptidion’s personal implementation of well-known search technique in the field of artificial intelligence (AI) called Monte Carlo tree search (MCTS) (Browne et al. 2012).

It is a probabilistic and heuristic driven search algorithm that combines the classic tree search implementations alongside machine learning principles of reinforcement learning. The top 30 sequences are used for docking and molecular dynamics. Among the final pool of five aptamer sequences, one was selected for the best features, converted into DNA, synthesized and experimentally tested to verify the interaction with the protein target.

### Contact angle measures

The static contact angle was measured using a home-made system. Before incubation electrodes were washed in the buffer solution to prepare the surface. For each measurement, 2 µL of deionized water was placed on the electrode without modifications or after aptamer-TAMRA deposition at different concentrations and washing steps. The images were acquired with a CMOS camera and analyzed by Drop-Analysis (Stalder et al. 2006). For each sample, due to the small size of sensing area, one single drop was taken. The results were reported as average value of left and right angle, and the errors were estimated as the standard deviations.

### Fluorescence microscopy measurements

Fluorescence microscopy images were taken using a Leica DMLA microscope (Leica Microsystems), equipped with a mercury lamp and fluorescence filter N2.1 (Leica Microsystems, Germany). All samples were observed with a × 10 magnification objective and measured via cooled CCD camera (DFC 420C, Leica Microsystems, Germany). Images were analyzed with Fiji software (Schindelin et al. 2012). The fluorescence of the solutions containing the aptamer-TAMRA was measured with a SPEX FluoroMax spectrofluorometer (SPEX Industries, Edison, NJ) using an excitation wavelength of 550 nm and recording the emission spectrum from 560 to 700 nm. The calibration curve was obtained measuring known amount of fluorescent aptamer-TAMRA, integrating the signal within the range of wavelength 560–580 nm. For unknown solutions, the integrated area value was converted to nanograms (ng) using the calibration curve reported in Fig. S1. The interaction aptamer–protein was also tested by using the fluorescence signal of TAMRA bounded to aptamer chain by incubation in the presence of the spike protein at increasing concentrations. The aptamer-TAMRA was deposed at 1 µM concentration in TRIS buffer on three different electrodes, in two independent experiments. The fluorescence signal was measured in solution after the addition of the spike protein (in PBS buffer) and 30 min incubation. The values were converted in nanograms of released aptamer using the calibration curves (Fig. S1).

### Electrochemical measurements

#### Aptamer immobilization

A total of 12 µL aptamer solution (AP_7462DNA without fluorophore 0.5 µM) in PBS 1X with 0.55 mM MgCl_2_ was drop casted on the carbon working electrode (CWE) of the screen-printed and incubated for 45 min at room temperature (RT) (25 °C). After washing twice with 500 µL PBS 1X containing 0.55 mM MgCl_2_, the screen-printed were dried under biological hood and used for tests using a Metrohm Potentiostat/Galvanostat/Impedance Analyzer (Origgio, VA, Italy).

#### Samples analyses

As positive controls, the spike protein at 0.001, 0.01, and 0.1 µM was used. A total of 12 µL of each spike concentration was added to the carbon electrodes on which it was previously deposed the aptamer at 0.5 µ M and incubated for 3 min at RT, washed with PBS buffer pH 7.4 1X and subjected to DPV analyses using 10 mM potassium hexacyanoferrate (II) (K_4_[Fe CN]_6_) in PBS 1X as chemical probe.

As negative controls were used: (i) bovine serum albumin (BSA) at 0.15 µM, 1.5 µM, 3 µM and 6 µM in PBS 1X; (ii) lysozyme at of 0.15 µM, 1.5 µM, 2 µM, 3 µM and 10 µM in PBS 1X.

A total of 12 µL of a solution of BSA or lysozyme was drop casted on the carbon WE of the screen-printed after the deposition of the aptamer at 0.5 µM and tested with differential pulse voltammetry (DPV).

A solution obtained by mixing the spike protein at 0.05 µM (positive sample) and BSA at 1.5 µM (negative sample) was also used. A total of 12 µL was deposed on the carbon screen-printed functionalized with the aptamer at 0.5 µM and tested using DPV. The same procedure was carried out using 12 µL of a solution containing the spike protein at 0.05 µM and lysozyme at 1.5 µM on the CWE with the aptamer at 0.5 µM.

### Voltammetry measurements

The DPV measurements were carried out using a 10 mM K_4_[Fe (CN)_6_] (Sigma-Aldrich) PBS 1X solution with a scan potential from − 0.2 to + 0.4 V at 0.01 V/s. The data were collected with NOVA 2.1.2 software and the anodic peak current was measured with the same software. For each measurement, the anodic peak current was recorded from the carbon electrode after deposition of the aptamer and considered the blank (i_pa_ Blank). The difference (Δi_pa_) between i_pa_ Sample and i_pa_ Blank obtained for each sample was calculated.

## Results

### Static contact angle

**Fig. 1:**
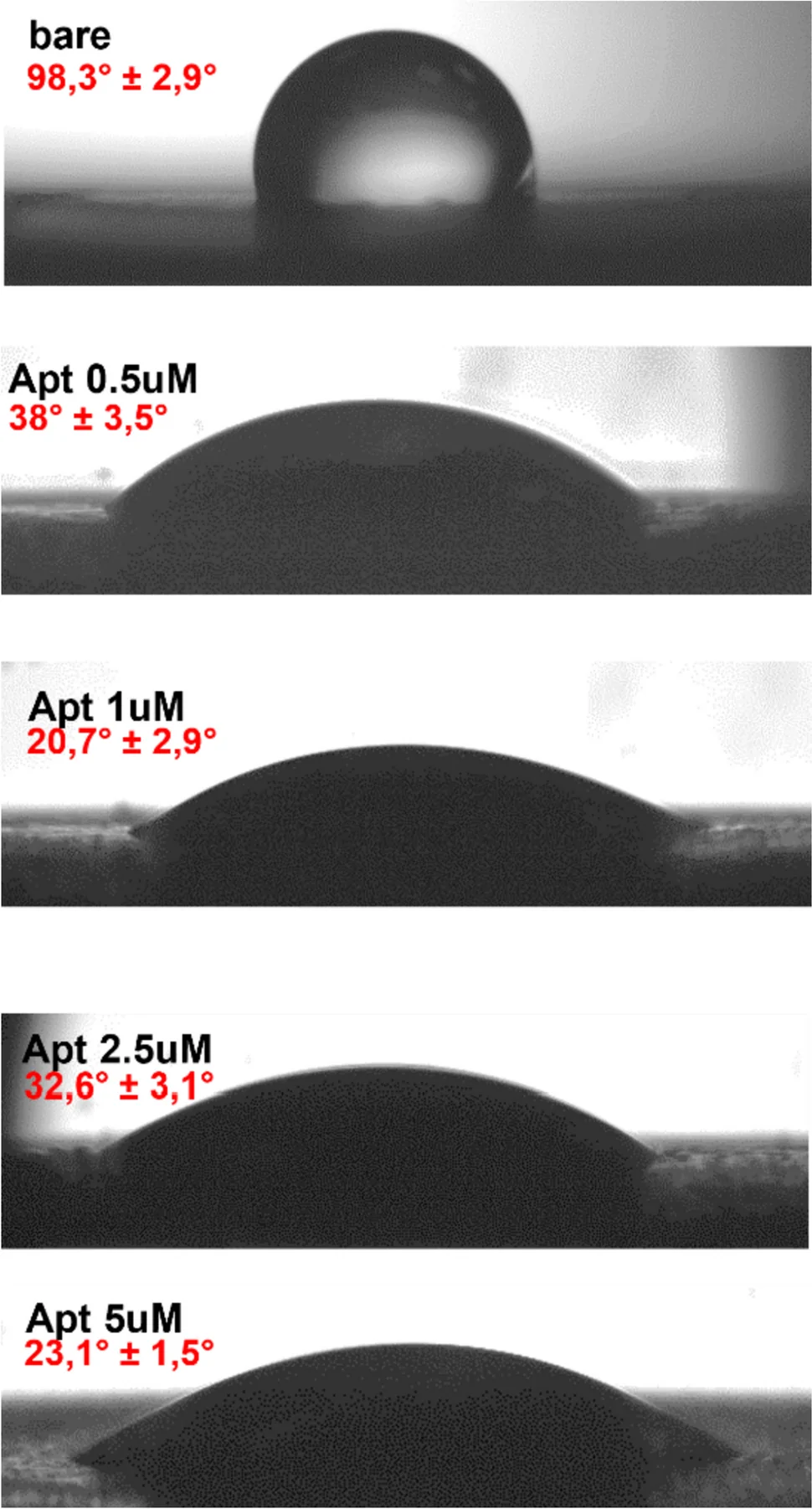
Contact angle analysis after incubation of 0.5 µM up to 5 µM of aptamer-TAMRA on electrode surface. Data are reported as mean value of left and right angle on the sample and the standard deviation

After aptamer-TAMRA incubation a clear surface modification was observed through contact angle analysis (Fig. 1).

The bare surface has a hydrophobic character as demonstrated by a contact angle value of about 100°; a clear diminish in the angle measured confirms a change to a more hydrophilic character as consequence of the negatively charged backbone of the aptamer-TAMRA immobilization. The variation in dependance of the concentration (from 0.5 up to 5 µM) is not statistically relevant and can be due to the variability of the electrode itself. These measurements together with the fluorescence characterization reported in the following paragraph suggest that a concentration of 0.5 µM was enough to cover the electrode surface.

## Fluorescence characterization

Two different buffers were used for the aptamer immobilization on the electrodes and for protein incubation and recovery with the aim to set up the best experimental conditions. Through the aptamer-TAMRA which has a fluorophore immobilized at 3′ end, we were able to monitor the aptamer-TAMRA binding and detaching from the carbon electrode surface, measuring in solution the fluorescence signal.

**Fig. 2:**
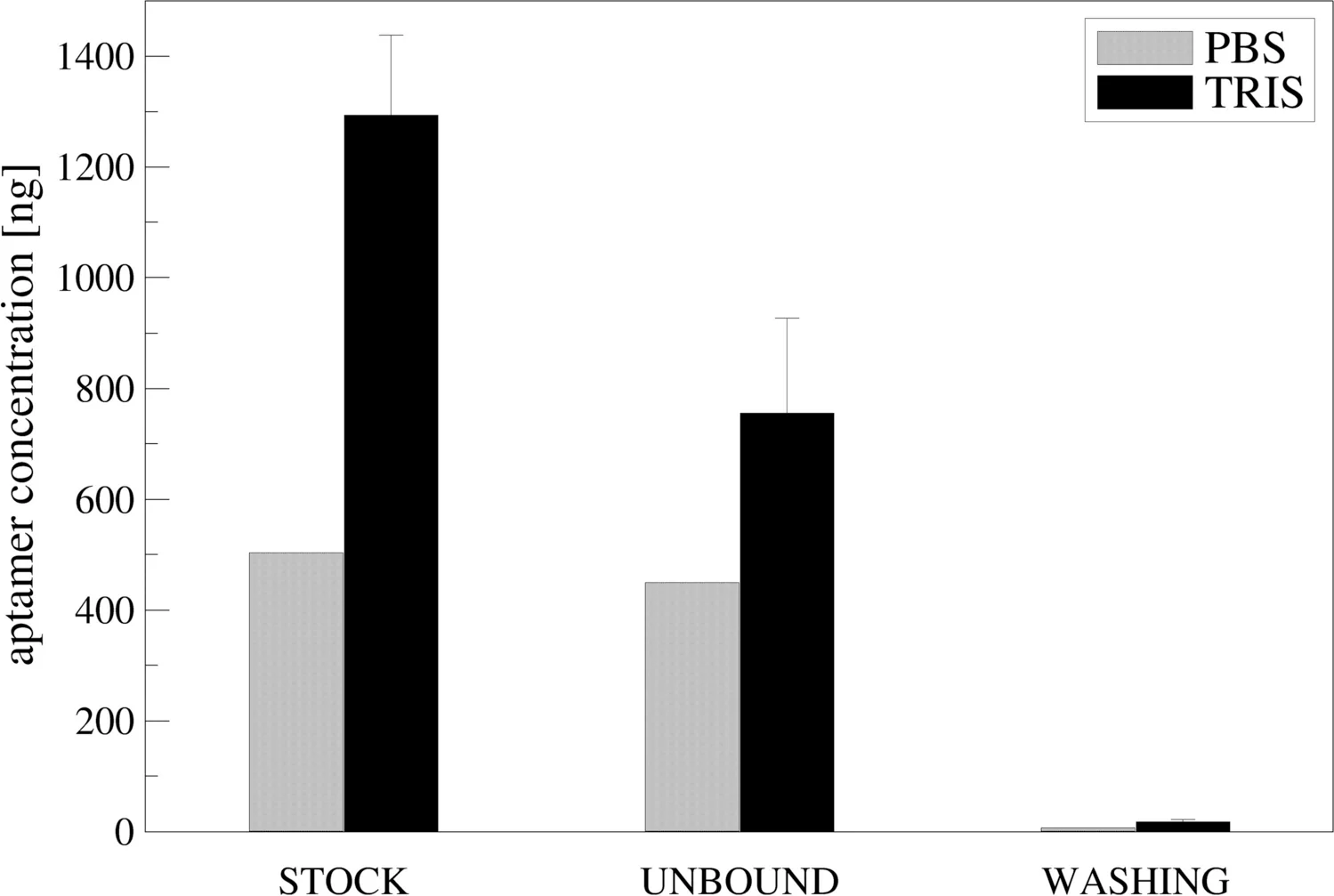
Quantification of aptamer by spectrofluorimetric evaluation in two different buffers using calibration curves reported in Fig. S1. Aptamer-TAMRA incubation was performed at 0.5 µM concentration in PBS 1X and 1 mM concentration in TRIS buffer

Figure 2 reports the aptamer concentration (ng) obtained by measuring the fluorescence signal of the solution (stock), the solution after 45 min incubation (unbound) and the solution after washing steps, reported as sum of the multiple washings (washing).

Comparing the buffers, it was possible to note that TRIS is more suitable for the aptamer binding since a significant low fluorescence signal was recorded in the unbound and washing solution (Fig. S2) respect to PBS buffer.

Increasing concentration, the aptamers-TAMRA were suspended in TRIS buffer and deposed on the carbon working electrodes, in order to select the concentration that could ensure an adequate coverage of the carbon electrode surface. The estimation of the aptamer concentration on the electrode was calculated as:

1 $$\mathrm{C}=\frac{\textit{FS}-\textit{FU}-\textit{FW}}{\mathrm{a}}$$

where *FS* is the fluorescence signal of the stock, *FU* is the fluorescence signal of the unbound, *FW* is the fluorescence signal of the washing and *a* is the regression coefficient determined by the calibration curve (Fig. S1).

**Fig. 3:**
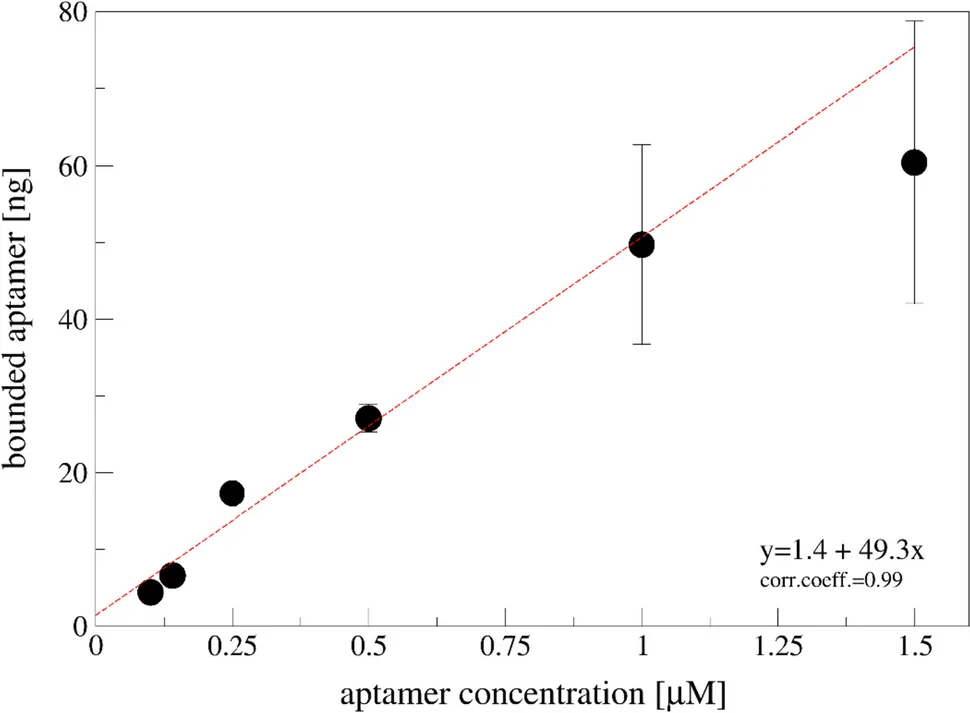
Quantification of the bounded aptamer in TRIS buffer, subtracting the fluorescence signal of unbounded and washing from stock solutions. Data are reported as mean of at least two different electrodes, and error bars represent the standard deviation. Linear regression was performed considering data up to 1 μM aptamer incubated on surface

Since concentrations higher than 1 µM do not change significatively the hydrophilicity of the surface, as reported in Fig. 1, we span a range between 0.1 and 1.5 µM, as shown in Fig. 3.

A linear increase up to 1 µM was observed, suggesting that higher concentrations cannot significantly modify the bounded aptamer amount.

**Fig. 4:**
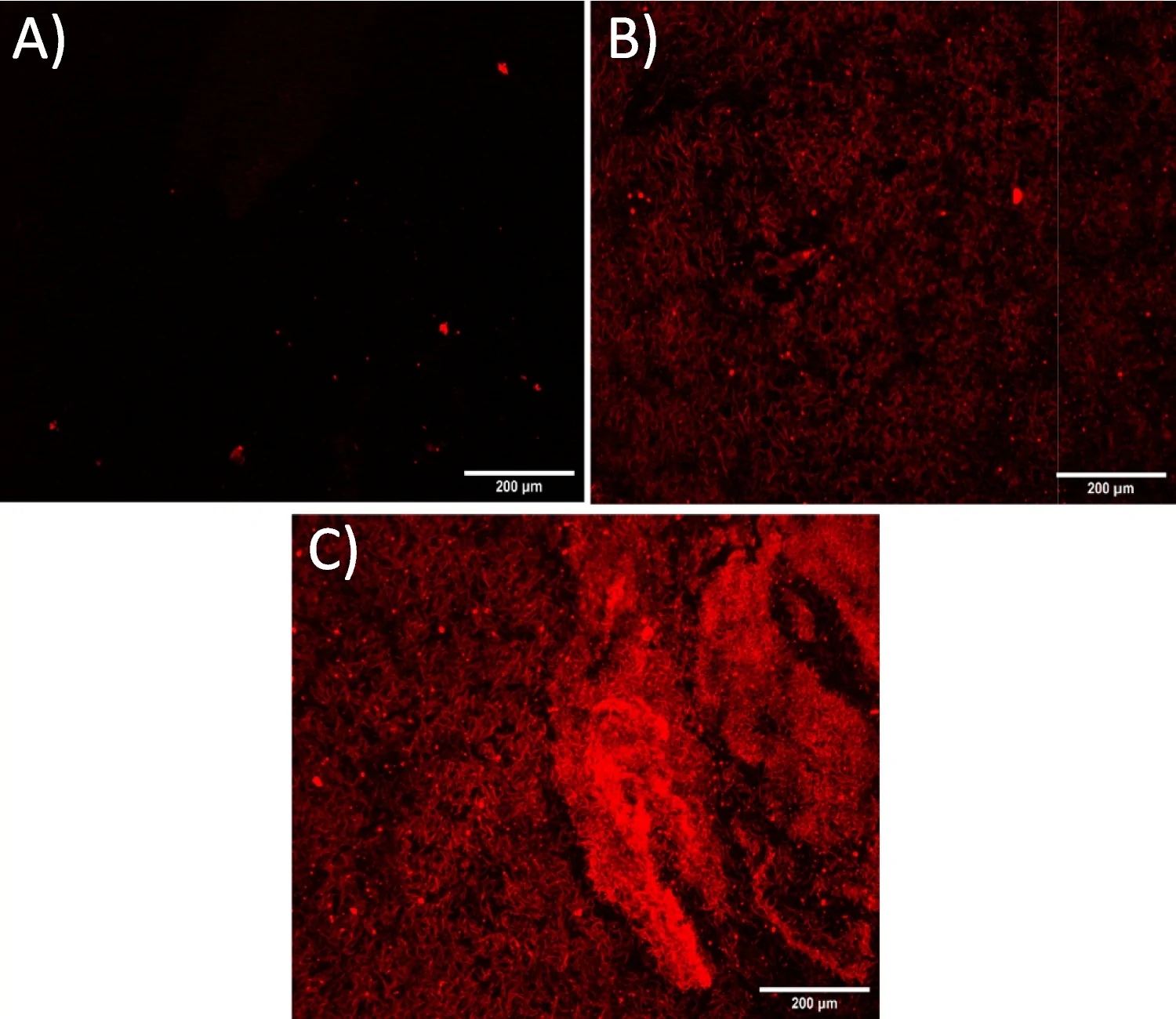
Fluorescent microscopy images of aptamer-TAMRA incubated on carbon electrodes at 0.1 µM (**A**), 0.5 µM (**B**), 1 µM (**C**). Images were acquired at × 10 magnification with 1 s of exposure time

A fluorescent microscopy evaluation was also performed to check the distribution and the homogeneity of the aptamers on the carbon electrode surface. Increasing the aptamer concentration, a fluorescence increase is recorded on the carbon electrode, as reported in Fig. 4.

The test performed with aptamer-TAMRA proves that at an increased concentration of the labeled aptamer from 0.1 (Fig. 4A) to 1 µM (Fig. 4B) corresponds to an increased detection of fluorescence on the surface of the electrode, causing however the aggregation at high concentration (Fig. 4C). From these experiments, a concentration of 0.5 µM of aptamer was therefore selected for the electrochemical measurements.

Moreover, the aptamer-TAMRA incubated with the presence of spike protein and BSA showed higher release of the aptamer for spike addition compared to BSA addition (Fig. S3).

## Electrochemical results

Electrochemical measurements have been performed using non fluorescent aptamer incubated at 0.5 µM concentration. The DPV conditions used are reported in Table 1.

**Table 1:** Differential pulse voltammetry values

Differential pulse voltammetry values	
Start potential	− 0.2 V
Stop potential	0.4 V
Step potential	0.005 V
Modulation amplitude	0.15 V
Modulation time	0.05 s
Interval time	0.5 s
Scan rate	0.01 V/s

The differences between the anodic peak current recorded before the addition of the sample (Ipa blank) and the anodic peak current recorded after addition of the sample (Ipa samples) are reported as Δ Ipa (expressed in µA) DPV.

**Fig. 5:**
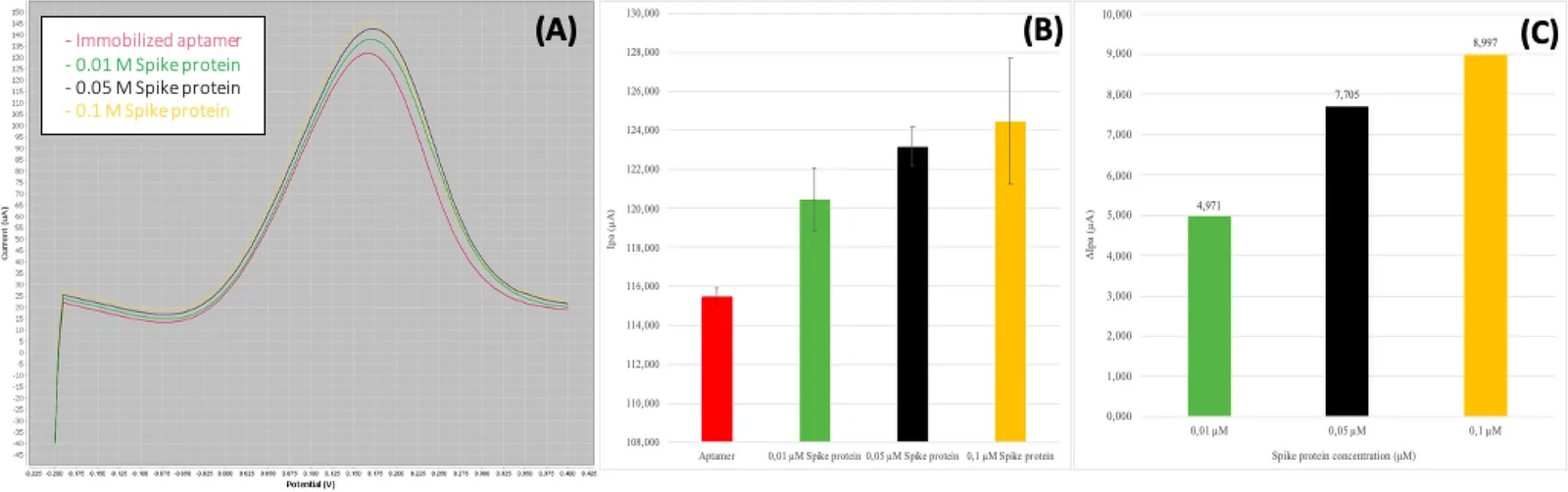
Results obtained from the differential pulse voltammetry (DPV) tests. **A** The original curves resulting after the addition of different spike concentrations (0.01 μM—0.05–0.1 µM) on the surface of the WE with the aptamer at 0.5 µM. **B** The Ipa values (µA) obtained after aptamer adsorption and after the addition of increasing concentrations of the spike protein. **C** The correspondent ∆Ipa values (µA) obtained after the addition of spike protein concentrations on the screen-printed electrode with the aptamer

The positive controls performed with the use of the spike protein reported in Fig. 5 indicate the expected detachment of the aptamer from the carbon surface, due to the higher affinity of the aptamer towards the spike compared to the carbon surface of the electrode. Moreover, the increase of the spike concentration corresponds an increased amount of aptamer detached from the electrode, proved by the increase of the positive values of the Δ Ipa measured.

**Fig. 6:**
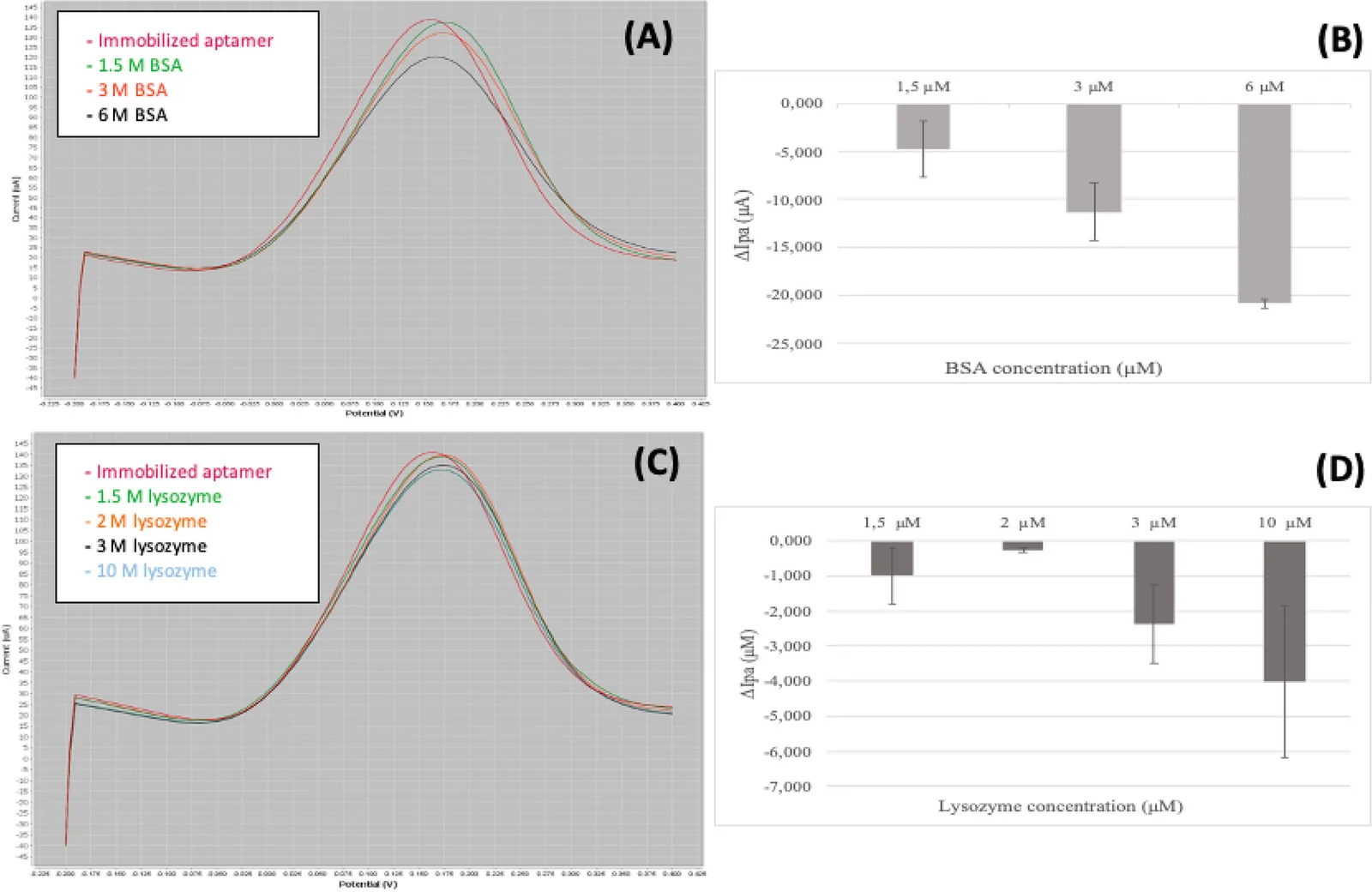
Curves and ΔIpa obtained for the tests performed with bovine serum albumine (BSA) and lysozyme. **A** Reports the original curves obtained using the modified SPE with the serial addition of Bovine Serum Albumine (BSA) at concentrations of 1.5 – 3 - 6 μM. **B** Shows the values of ΔIpa obtained for the BSA concentrations tested. **C** Shows the original curves of the deposition of increasing concentrations of lysozyme (1.5 – 2 – 3 - 10 μM). **D** Reports the values of ΔIpa obtained for the lysozyme concentrations tested

The experiments performed with BSA and lysozyme and used as negative controls are reported in Fig. 6. Carbon screen-printed 1 and 2 (indicated as chip 1 and chip 2) coated with the aptamer at 0.5 µM and incubated with BSA at 1.5 µM, 3 µM and 6 µM, and carbon screen-printed 3 and 4 (named chip 3 and chip 4), coated with the aptamer at 0.5 μM, incubated with lysozyme at 1.5 µM, 2 µM, 3 µM and 10 µM produced negative values of the Δ Ipa indicating that the aptamer did not detached from the carbon surface of the electrode.

This behaviour (negative Δ Ipa) proves that the aptamer has no affinity towards proteins different from the spike target protein, even when tested against two proteins present in saliva, a possible sample for the detection of the SARS-CoV-2 virus. Also, the increase of the BSA and lysozyme added to the chips produces more negative values of the Δ Ipa suggesting that the proteins lay on the electrodes covered with the aptamer producing a decrease of the signal.

Tests using the spike protein at 0.05 µM in the presence of both 0.15 µM BSA and 0.15 µM lysozyme were carried out with a standard deviation of 5201 (*n* = 3).

We have shown the affinity and the high sensitivity of the aptamer towards the spike protein, target of our tests, and the absence of affinity towards the most abundant proteins present in the saliva samples. Even if we do not perform all the control experiments, from our dataset, we can infer a high specificity of the aptamer sequence that is able to recognize the protein at low concentration respect to other two abundant proteins. We also tested the protein at 0.05 µM in the presence 0.15 µM BSA and 0.15 µM lysozyme, and the standard deviation obtained was 5201 (*n* = 3).

**Fig. 7:**
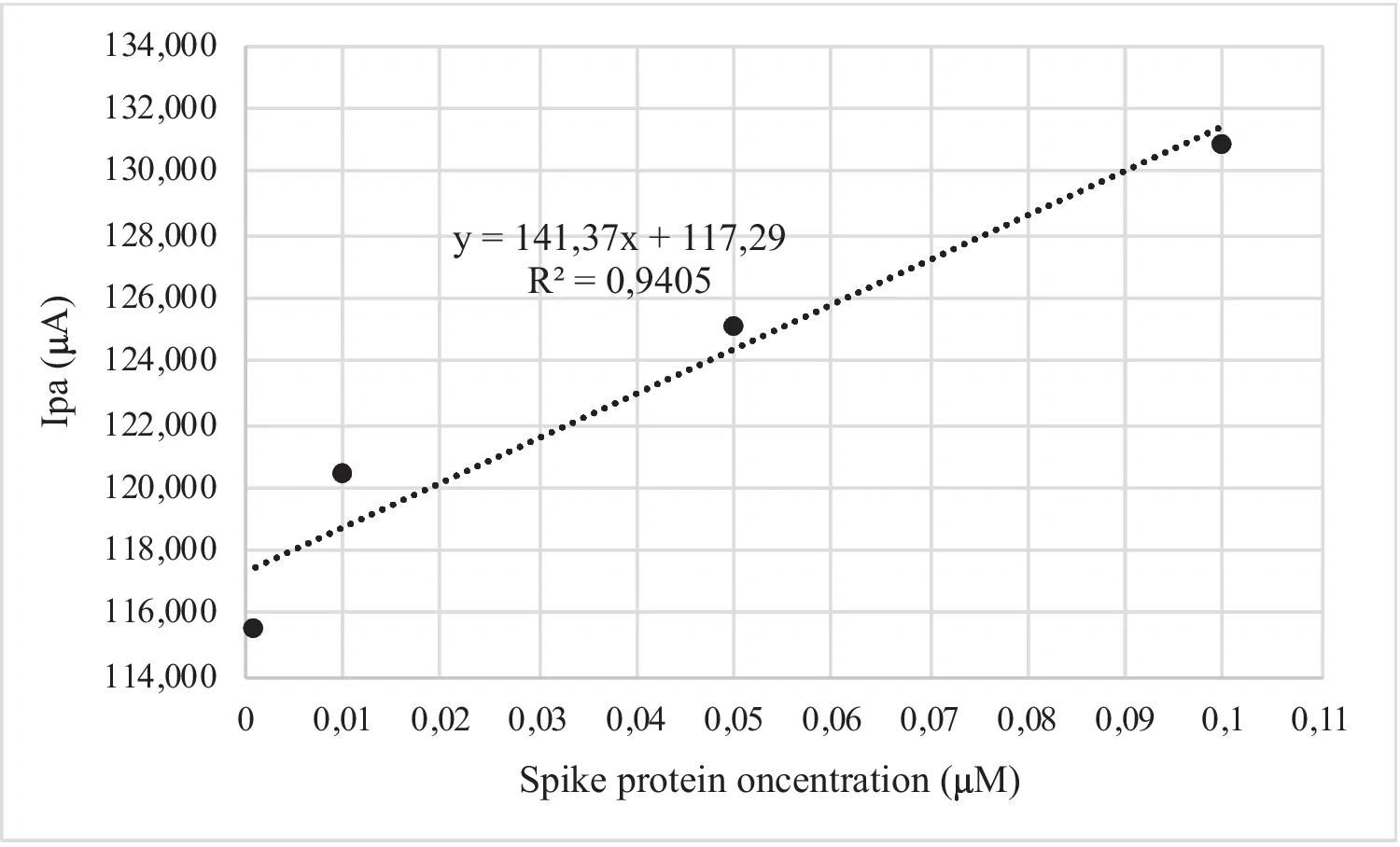
Linear range obtained for the carbon SPE used

The linear range of the carbon screen-printed electrode was calculated after the deposition of four different spike protein concentrations (0.001–0.01–0.05–0.1 µM) (Fig. 7). According to the R^2^ = 0.948, there is a significative correlation between the increasing concentration of the spike protein concentration and the decreasing values of Δ Ipa. The LOD (limit of detection) was obtained according to the following equation: LOD = 3•SD (of the blank)/ slope of calibration curve. It is 0.033 µM.

## Discussion

**Fig. 8:**
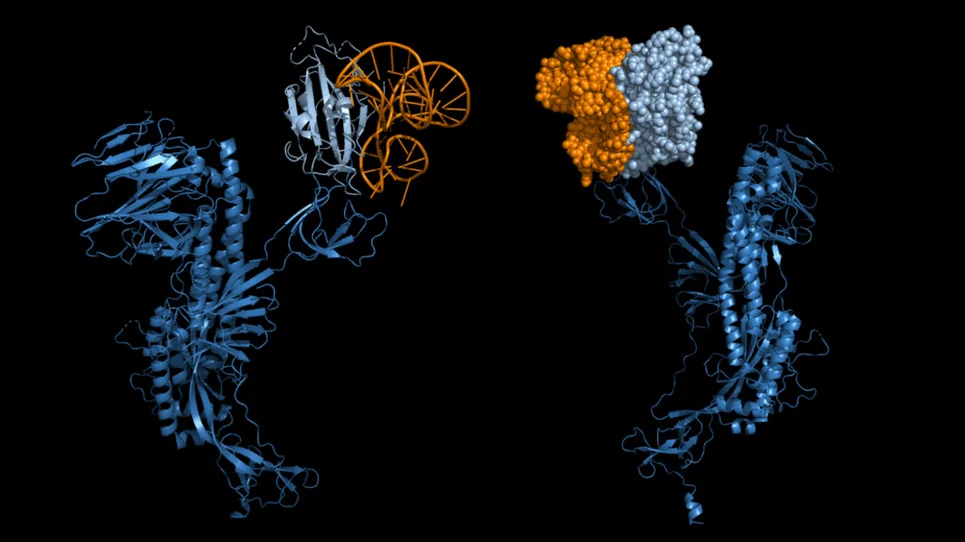
Image of the results of the aptamer designed with docking using the in silico design. The structure reported is obtained from the sequence selected by the programs used

In this work, we evaluated the ability of a computational designed aptamer sequence to recognize its target using an easy electrode modification based on the adsorption of the aptamer sequences onto the carbon surface, taking advantage of the interaction between the DNA bases and carbon-based materials through ππ stacking forces, as highlighted by Eissa and co-workers (Eissa et al. 2019) (Fig. 8).

The interaction between the aromatic rings of the nucleobases of DNA aptamer with the carbon-based electrodes is a well-known process that has been used for developing biosensors (Song et al. 2012a, b; Sen et al. 2022). Due to the stronger forces (Van der Waals, hydrogen bond and electrostatic) which take place between the aptamer and its target (spike protein), after the addition of the spike protein on the carbon electrode, the aptamer desorbs from the electrode surface and binds its target in solution producing an increase in the peak value obtained by the electrochemical measurement.

The electrode surface modification obtained by the absorption of the aptamer is highlighted by the change in the contact angle measurement (Fig. 1). The increase in the hydrophilicity due to the presence of negatively charged phosphodiester backbone of DNA is also observed by other authors using different immobilization strategies (Alnaimi et al. 2022; Bagheryan et al. 2016; Kaur et al. 2019) finding similar values. This measure is indicative of a modification of the surface from a morphological and chemical point of view, resulting in a more hydrophilic surface independently on the aptamer concentration used.

To optimize the adsorption of the aptamer on carbon modified electrode, we tested two different buffers, PBS buffer and TRIS buffer, finding better adsorption when TRIS buffer is used (Fig. 2). The higher salt content in the TRIS buffer is probably responsible for the higher adsorption on carbon-based materials: the same buffer composition is also used by Eissa and co-workers (Eissa et al. 2019) on different carbon-based materials. Using TRIS buffer, we observed a linear increase up to 1 µM of incubation solution (Fig. 3), and higher aptamer concentrations do not increase significantly the amount of bounded aptamer suggesting that a saturation of the electrode surface is reached. Similar aptamer concentration has been used to saturate the electrode surface even using different chemistry (Alnaimi et al. 2022; Bagheryan et al. 2016; Eissa et al. 2019).

The aptamer sequence labeled with TAMRA proved the adsorption of the aptamer on to the electrode surface by using the fluorescence microscopy (Fig. 4) up to 1 µM, while higher aptamer solution resulted in lower fluorescent signal (data not shown) due to the well-reported quenching effect caused by carbon-based materials (Liao et al. 2017; Karimi and Dabbagh 2019; Khan et al. 2020). Since at 1 µM aptamer the solution showed aggregates on the electrode surface, a concentration of 0.5 µM was chosen for the electrochemical experiments. Monitoring the anodic peak current variations (Fig. 5), it was possible to confirm the aptamer detachment from the carbon electrode surface, while in Fig. 6, it is shown the specificity for the spike protein. In fact, using bovine serum albumin (BSA) and lysozyme, proteins present in the medium (saliva) are hypothesized as sample to test for the SARS-CoV-2 virus; no match was detected by electrochemistry assays. Figure 6 shows the positive values of the peaks for the target proteins and the negative peaks obtained for the other two proteins tested, indicating that no complex between the aptamer and BSA and between aptamer and lysozyme was observed. The strategy of detecting the target through the detachment of the sequence from the surface has also been used in fluorescence-based aptasensors (Khan et al. 2020; Liao et al. 2017); however, the electrochemical measurement is more sensitive and more easily integrable in portable platforms. The electrochemical method reported to test the target binding and the specificity of the developed aptamer sequence (a comparison with a standard fluorescence detection is reported in Figure S3) was selected for the sensitivity and possibility to build a portable system.

Several configurations both on measurements type and aptamer immobilization strategies have been used to develop efficient electrochemical aptasensors (Radi and Abd-Ellatief 2021; Topkaya and Cetin 2021). Electrochemical aptasensor application can be found in different fields from cancer biomarkers (Negahdary et al. 2019) to food contaminants (Wang et al. 2020) up to whole cells (Ziółkowski et al. 2021): examples of the different electrode configurations include a simple gold coated electrode (Chen and Guo 2013) or more complex systems like gold nanowires or nanoroads (Husna et al. 2023), carbon nanotubes or multiwall (Liu et al. 2010; Khan et al. 2018, Rezaei et al. 2018; He et al. 2020; Alnaimi et al. 2022), graphene (Kaur et al. 2019) or diamond (Asai et al. 2020). Here, we present a simple way to check the goodness of the aptamer sequence that has been set up using a computational tool allowing to reduce the time to 3 weeks compared to the classical method named SELEX (systemic evolution of ligands by exponential enrichment) which takes about 3–4 months and is more expensive.

We designed a machine learning approach that generates potential specific RNA-aptamers for target proteins based on a discriminative deep learning classifier. For the classification model, input aptamer and protein sequences were encoded on the fly. The optimal combination chosen of encoding parameters yielded 1500–2000 features for both aptamer and protein sequence.

Different approaches have been proposed using molecular dynamics (Navien et al. 2021) or machine learning (Bashir et al. 2021) whose efficacy of these approaches has been recently reviewed by many authors (Chen et al. 2021; Buglak et al. 2020; Khoshbin et al. 2021) and confirmed the value of our work. The possibility to obtain a specific sequence is very important for the detection of spike protein which rate of mutation is high, as showed by the last mutation detected last year (BA2.86 (Pirola), EG.5 (Eris), XBB.1.5 (Kraken), and XBB.1.16 (Arcturus), FL.1.5.1 (Fornax), BA.2.75 (under monitoring)). It can make possible to detect the mutations and to be ready with the new test in 1 month.

The good response obtained with DPV using the aptamer designed by the bioinformatic platform APTERION allowed to think that aptamers are useful for the construction of an electrochemical biosensor which can respond to specificity and rapidity requirements and that electrochemistry assays are useful for testing aptamers’ specificity and sensitivity.

## Supplementary Information

Below is the link to the electronic supplementary material.

Supplementary file1 (PDF 387 KB)

## Data Availability

All data generated or analyzed during this study are included in this published article (and its supplementary information files). All electrochemical data were produced at the University of Udine laboratory (Italy); Supplementary material originals and Figs. 1, 2, 3, and 4 were produced at Indivenire Srl (Trento, Italy).
