# Supplementary material for: Computational aptamer design for spike glycoprotein (S) (SARS CoV-2) detection with an electrochemical aptasensor
Source: Appl Microbiol Biotechnol. 2024 Mar 12;108:259. doi: 10.1007/s00253-024-13066-w (PMC10933206; doi:10.1007/s00253-024-13066-w)
Supplement: Supplementary file 1 [file 253_2024_13066_MOESM1_ESM.pdf]

SUPPLEMENTARY INFORMATION AMBB-D-23-00029R2

Manuscript:
Computational aptamer design for spike glycoprotein (S) (SARS CoV-2) detection with an electrochemical aptasensor

Journal: Applied Microbiology and Biotechnology

Authors:
Alessia Cossettini[1], Laura Pasquardini[2], Antonello Romani[3], Aldo Feriani[3], Debora Pinamonti[1], Marisa Manzano1*

[1]Department of Agriculture Food Environmental and Animal Sciences, University of Udine, Italy
[2]Indivenire srl, via alla Cascata 56/C, 38123 Trento, Italy
[3]Arta Peptidion srls, via Quasimodo 11, 43126 Parma, Italy

Corresponding author:
Marisa Manzano; marisa.manzano@uniud.it; Dipartimento di Scienze AgroAlimentari, Ambientali e Animali, via Sondrio 2/A, 33100 Udine, Italia
phone: +390432558127
fax: +390432558130

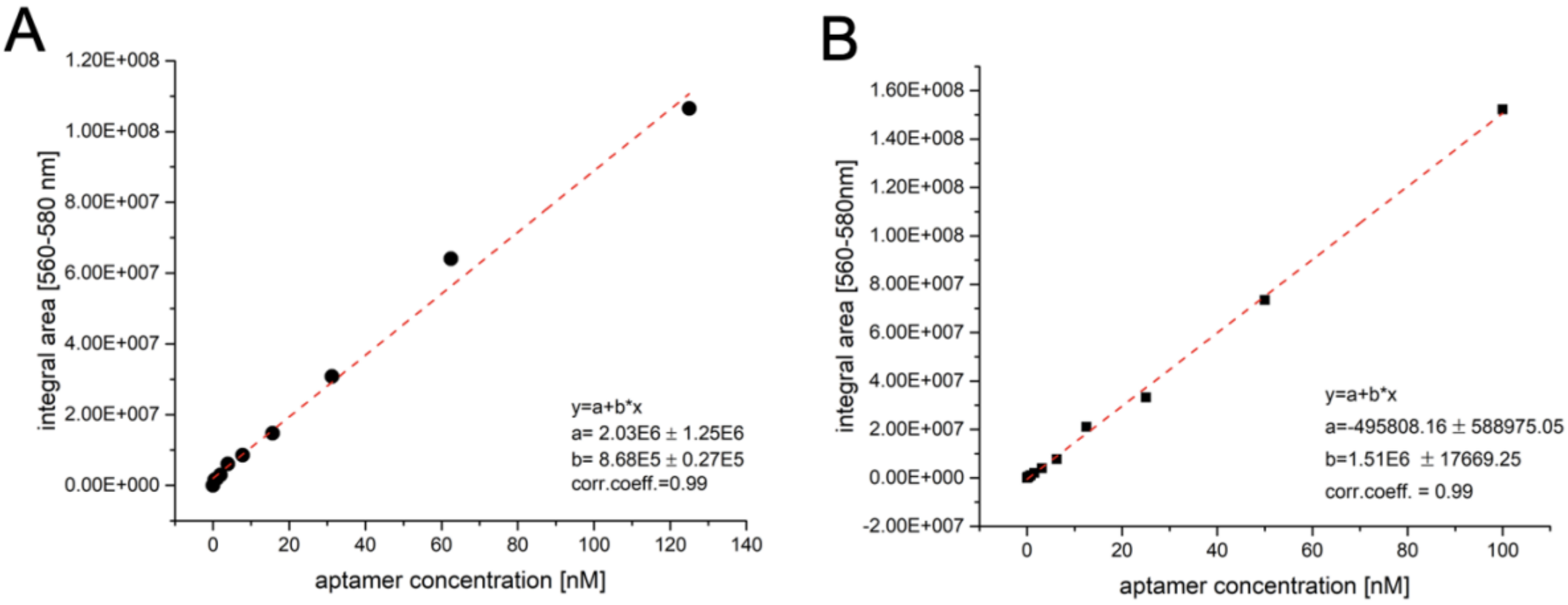

Fig. S1 Calibration curve of aptamer-TAMRA in PBS buffer (A) or TRIS buffer (B). Linear regression on short concentration range is reported in red dashed lines.

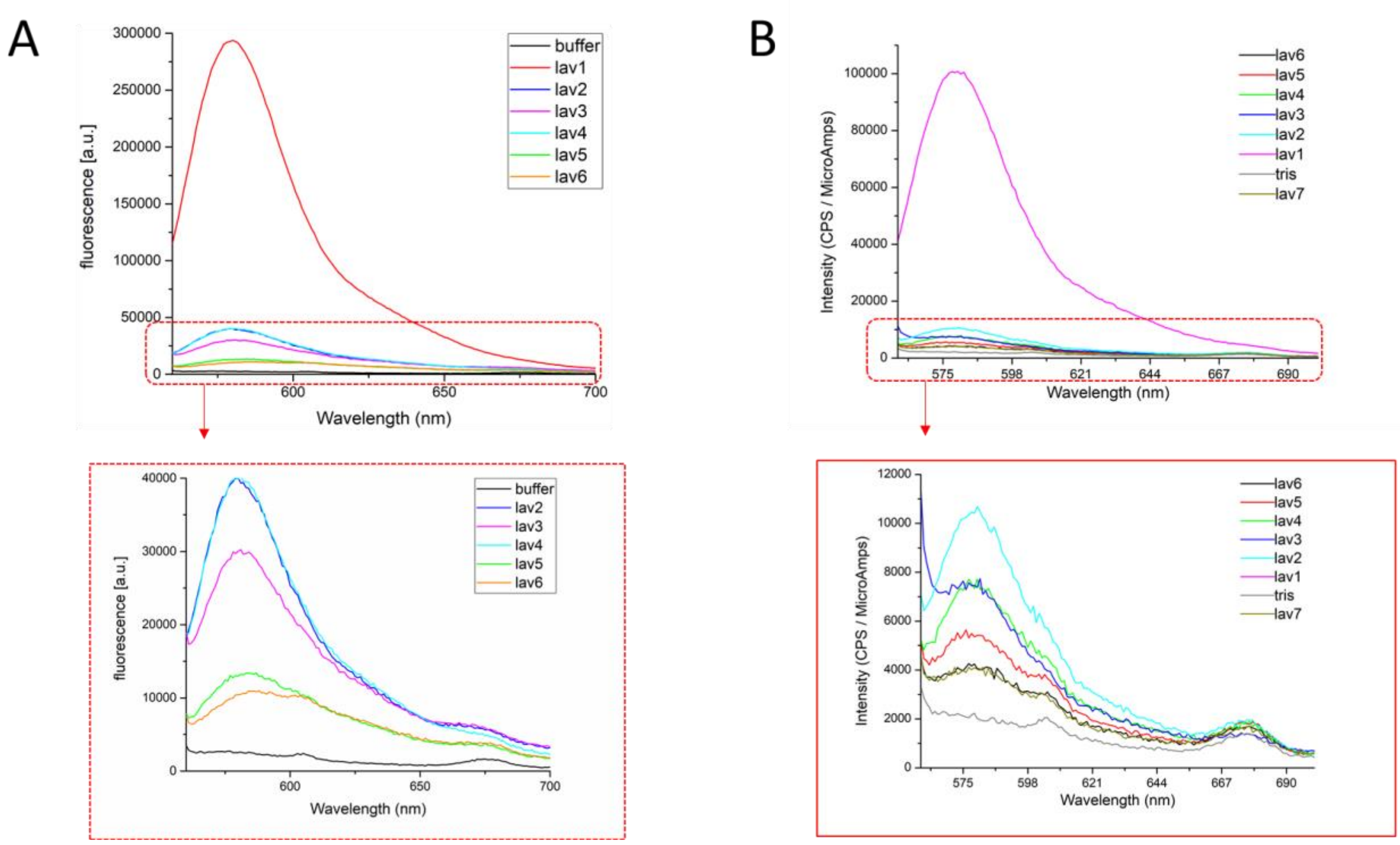

Figure S2: Spectrofluorimetric analysis on washing steps performed in PBS buffer (A) or TRIS buffer (B). A plot magnification is reported below, highlighting washing 2 up to 6 or 7.

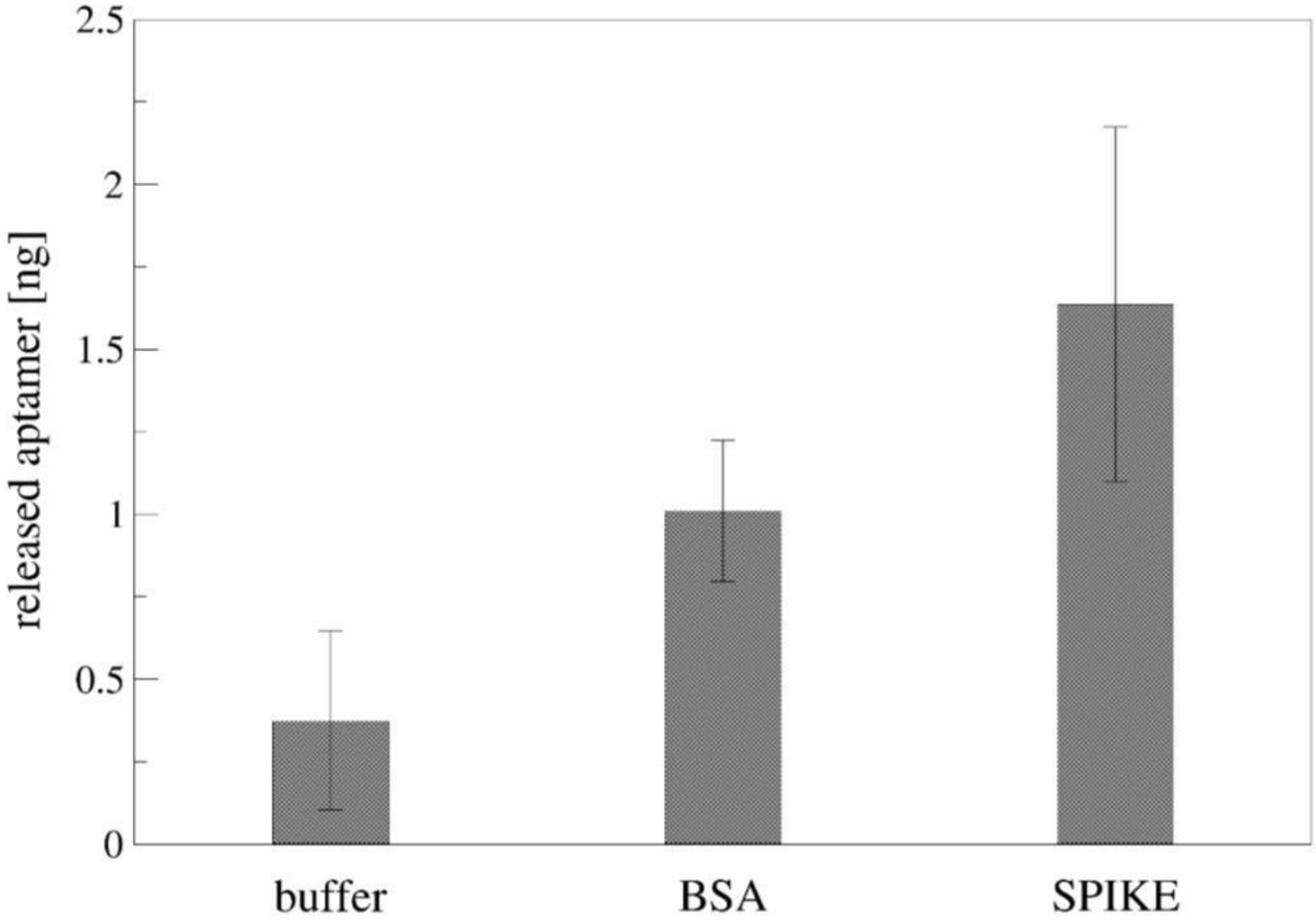

Fig. S3 fluorescent aptamer quantification after released from electrode caused by protein interaction. Bars represent the mean value of two independent experiments and error bars represent the standard deviation.
